# ANGPT2/Tie2 Enhances H3K18la‐Mediated Macrophage M2 Polarization to Promote Endothelial Cell Proliferation in the Chronically Ischaemic Brain

**DOI:** 10.1002/cns.70879

**Published:** 2026-04-12

**Authors:** Chuyang Tai, Cong Ling, Yang Yang, Ni Mo, Cian Yao, Songtian Lv, Baoyu Zhang, Hui Wang, Chuan Chen

**Affiliations:** ^1^ Department of Neurosurgery Third Affiliated Hospital of Sun Yat‐Sen University Guangzhou Guangdong China; ^2^ Department of Radiology Third Affiliated Hospital of Sun Yat‐Sen University Guangzhou Guangdong China

**Keywords:** angiogenesis, encephalomyosynangiosis, endothelial cell, histone H3 lysine 18 lactylation, macrophage polarization, Tie2‐expressing monocytes/macrophages

## Abstract

**Aims:**

This study aimed to investigate the specific mechanism by which angiopoietin‐2 (ANGPT2)/Tie2 signaling in macrophages promotes endothelial cell (EC) proliferation in the chronically ischaemic brain (CIB).

**Methods:**

We first analyzed the polarization status of primary Tie2‐expressing macrophages (TEMs) and Tie2‐overexpressing THP‐1‐derived macrophages (Tie2‐TDMs) following ANGPT2 treatment and detected the expression of representative proangiogenic factors. Subsequently, lysine lactylation (Kla) levels were measured, and chromatin immunoprecipitation (ChIP) assays were performed to explore the downstream activity of ANGPT2/Tie2 signaling. Additionally, in vitro functional assays using human umbilical vein endothelial cells (HUVECs) and in vivo experiments in a rat model of chronic cerebral ischaemia were conducted to confirm the effect of ANGPT2/Tie2‐regulated macrophages on angiogenesis.

**Results:**

In response to ANGPT2 treatment, the expression of M2 polarization markers and proangiogenic factors increased in TEMs and Tie2‐TDMs. Concurrently, LDHA and H3K18la were elevated, and ChIP assays confirmed the regulatory role of ANGPT2/Tie2 signaling in H3K18la‐mediated transcriptional regulation. The viability of HUVECs cocultured with Tie2‐TDMs was increased. Finally, ANGPT2 overexpression increased M2‐polarized TEM infiltration in the CIB; additionally, rats injected with ANGPT2‐pretreated TEMs exhibited more prominent EC proliferation.

**Conclusion:**

ANGPT2/Tie2 induces the H3K18la‐mediated M2 polarization of macrophages to facilitate EC proliferation and angiogenesis in the CIB.

## Graphical abstract

ANGPT2/Tie2 signaling upregulates LDHA and increases H3K18la, promoting the polarization of macrophages towards an M2 phenotype. Such M2 macrophages secrete high levels of proangiogenic factors, which facilitate endothelial cell proliferation and angiogenesis in the chronically ischaemic brain.

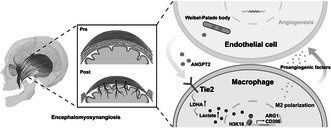

## 1 Introduction

Chronically ischaemic cerebrovascular disease (CICD) is the primary cause of stroke, which frequently results in severe disability or even mortality [1, 2]. Unfortunately, revascularization surgery fails to meet the expectations in approximately half of CICD patients because of an insufficient angiogenic potential [3].

Angiopoietins (ANGPTs) constitute a key family of regulators of angiogenesis across diverse ischaemic contexts [4, 5]. As a critical member, ANGPT2 competes with ANGPT1 for binding to Tie2 (also known as the receptor tyrosine kinase TEK), thereby promoting endothelial cell (EC) proliferation [6, 7, 8]. Notably, recent studies have shown that ANGPT2 also recruits Tie2‐expressing monocytes/macrophages (TEMs) to ischaemic tissues, where these cells secrete high levels of proangiogenic factors—a function that has been reported in the chronically ischaemic brain (CIB) [9, 10, 11, 12]. However, the specific regulatory mechanism underlying the secretion of proangiogenic factors by TEMs remains unclear.

As a subset of macrophages, the functions of TEMs are tightly coupled to their polarization status [13]. In the tumor microenvironment, TEMs are classified as a type of tumor‐associated macrophage (TAM) and are considered M2‐like macrophages because of their high expression of proangiogenic factors [10, 14]. This unique phenotypic trait is likely conferred by their expression of Tie2—a feature that distinguishes them from conventional macrophage populations.

Histone lactylation, a newly characterized posttranslational modification, modulates gene expression by altering chromatin accessibility [15, 16]. In hypoxic microenvironments, increased glycolysis leads to intracellular lactate accumulation [17, 18]. This lactate not only serves as a metabolic intermediate but also acts as a substrate for lactylation. Notably, lysine lactylation (Kla) at multiple histone loci has been shown to participate in the regulation of macrophage polarization [15, 19, 20]. Lactylation modification of multiple lysine sites on histones has been shown to enrich promoter regions of M2‐associated genes (e.g., *ARG1* and *CD206*), thereby driving macrophages towards a repair‐promoting phenotype [21, 22, 23].

Based on the information above, we hypothesize that the ANGPT2/Tie2 axis mediates macrophage M2 polarization through the regulation of histone lactylation and subsequently promotes EC proliferation in the CIB. In this study, primary TEMs and Tie2‐overexpressing macrophage lines were treated with exogenous ANGPT2 to validate the effect of the ANGPT2/Tie2 axis on histone lactylation and M2 macrophage polarization and identified histone H3 lysine 18 lactylation (H3K18la) as a key regulatory modification. Additionally, chromatin immunoprecipitation (ChIP) assays were performed to detect the enrichment of H3K18la at the promoters of M2‐associated genes following an ANGPT2 pretreatment. Furthermore, the effects of this mechanism on EC proliferation were explored through in vitro hypoxic coculture experiments involving Tie2‐overexpressing macrophages and human umbilical vein endothelial cells (HUVECs) and in vivo experiments with rats with chronic cerebral ischaemia subjected to the overexpression of ANGPT2 or the injection of ANGPT2‐pretreated TEMs. We anticipate that our findings will provide novel insights into both the treatment of CICD and research on angiogenesis.

## 2 Methods

### 2.1 Ethics

Studies involving humans were performed according to the Declaration of Helsinki (2000) and received approval from the Ethics Committee of the Third Affiliated Hospital of Sun Yat‐sen University (No. RG2025‐225‐02). All participants provided written informed consent. The animal experiments strictly followed the ARRIVE guidelines and were approved by the Animal Ethics Committee of Sun Yat‐sen University (No. IACUC‐202501‐4).

### 2.2 Primary TEMs Sorting

Peripheral blood mononuclear cells (PBMCs) were isolated from the peripheral blood of CICD patients using human whole blood mononuclear cell separation solution (Solarbio, China). Then, CD14^+^ cells were isolated via magnetic‐activated cell sorting (MACS; Miltenyi Biotec, Germany) using human CD14 magnetic beads (Miltenyi Biotec, Germany). TEMs and Tie2‐negative monocytes/macrophages (TNMs) were sorted via MACS using an anti‐Tie2 (ab221154, Abcam, UK) antibody labeled with NHS‐Biotin (Thermo Fisher, USA) and MicroBeads (Miltenyi Biotec, Germany). Flow cytometry and immunofluorescence staining were performed to identify the cellular phenotypes. TEMs and TNMs were stimulated with 25 ng/mL M‐CSF for 24 h to induce their differentiation into macrophages (Figure S1).

### 2.3 Cell Lines

The human monocytic leukemia cell line THP‐1 and HUVECs were purchased from American Type Culture Collection (ATCC). THP‐1 cells were cultured in Roswell Park Memorial Institute (RPMI) 1640 medium (Gibco, USA), and HUVECs were cultured in high‐glucose Dulbecco's modified Eagle's medium (DMEM) (Gibco, USA), each supplemented with 10% foetal bovine serum (FBS) and 1% penicillin–streptomycin (Gibco, USA). Cells were routinely cultured in a humidified incubator at 37°C with 21% O_2_ and 5% CO_2_. For hypoxic culture, cells were cultured in a tri‐gas incubator with 1% O_2_, 94% N_2_, and 5% CO_2_. Subsequent experiments were conducted with HUVECs between passages 3 and 6.

### 2.4 Tie2 Overexpression in THP‐1‐Derived Macrophages (TDMs)

A lentivirus for Tie2 expression (LV‐Tie2) and the corresponding negative control (LV‐NC) were constructed and packaged by ReGene Biotechnology Co. Ltd. (Guangzhou, China). THP‐1 cells were infected with LV‐Tie2 in the presence of 5 μg/mL polybrene (Sigma, Germany) for 24 h, after which routine culture was resumed. Stable transfectants were selected with 2 μg/mL puromycin (MedChemExpress, USA) starting at 72 h postinfection. The infection efficiency was assessed using fluorescence microscopy and qPCR. TDMs were obtained by exposing THP‐1 cells to 100 ng/mL phorbol 12‐myristate 13‐acetate (PMA) for 24 h (Figure S2).

### 2.5 ANGPT2 Treatment

In accordance with previous studies, primary TEMs and TNMs were treated with 100 ng/mL ANGPT2 protein (HY‐P7510; MedChemExpress, USA) for 3 h, while TDMs were exposed to the same concentration of ANGPT2 for 6 h.

### 2.6 Flow Cytometry Analysis

Flow cytometry was used to quantify macrophage polarization after ANGPT2 treatment. Cells (2 × 10^6^) were transferred to flow cytometry tubes and blocked with Human TruStain FcX (BioLegend, USA) for 15 min at room temperature. The cells were then stained for 1 h on ice with anti‐CD80‐PE (E‐AB‐F1232D, Elabscience, China), anti‐CD206‐PerCP/Cyanine5.5 (321,121, BioLegend, USA), anti‐iNOs‐AF488 (53‐5920‐80, Invitrogen, USA), and anti‐ARG1‐APC (17‐3697‐82, Invitrogen, USA) antibodies. After two washes with cold phosphate‐buffered saline (PBS), the cells were resuspended in 200 μL of PBS and analyzed using a flow cytometer (BD Biosciences, USA). The data were analyzed with FlowJo v10 (Tree Star, USA). Each experiment was performed at least three times.

### 2.7 Quantitative Real‐Time PCR (qRT‐PCR)

Total RNA extraction from cells was carried out using TRIzol reagent (Thermo Fisher, USA) in strict accordance with the manufacturer's standard protocols to ensure the integrity and purity of the isolated RNA. Subsequent first‐strand complementary deoxyribonucleic acid (cDNA) synthesis was performed via reverse transcription (RT) reaction using a PrimeScript II 1st Strand cDNA Synthesis Kit (Takara Bio Inc., China), which facilitates efficient conversion of RNA templates into cDNA with high fidelity. Quantitative real‐time PCR amplification reactions were performed on the ABI QuantStudio 5 Real‐Time PCR System (Thermo Fisher, USA) and UltraSYBR Green Mixture (CWBIO Co. Ltd., China) was used in the reaction mixture. The sequences of the primers are listed in Table S1.

### 2.8 Determination of the Intracellular l‐Lactate Concentration

The intracellular l‐lactate concentration was measured using an l‐lactate assay kit (Beyotime, China). Cells were lysed with BeyoLysis Buffer A for metabolic assays, followed by centrifugation at 12,000 × *g* for 10 min. The supernatant was incubated with WST‐8 Working Solution for 30 min, after which the absorbance at 450 nm was measured using a microplate reader (Thermo Fisher, USA). The l‐lactate concentration was calculated from the standard curve. Each experiment was performed at least three times.

### 2.9 Chromatin Immunoprecipitation (ChIP) Assay

A ChIP assay was performed to verify the regulatory effect of ANGPT2/Tie2 on H3K18la‐mediated transcriptional regulation. TDMs were fixed with 1% formaldehyde and lysed with SDS lysis buffer (Beyotime, China). Next, the cells underwent sonication, followed by heat treatment at 65°C to reverse the protein–DNA crosslinks. After being incubated with Protein A + G Agarose/Salmon Sperm DNA (Sigma, Germany), the samples were further incubated overnight at 4°C with either 5 μg of anti‐H3K18la (PTM‐1427RM, PTM BIO, China) or 5 μg of anti‐IgG (30000‐0‐AP, Proteintech, China). After the precipitates were washed, the associated DNA was purified and concentrated. Finally, qRT‐PCR was performed to analyze the DNA fractions, and relative gene expression was calculated as the fold change relative to input DNA and normalized to a sample pretreated with PBS and IP with anti‐H3K18la as a reference. Details of the primers used are available in Table S2.

### 2.10 Cell Counting Kit‐8 (CCK‐8) Assay

A CCK‐8 assay was performed to evaluate the viability of HUVECs. Briefly, 1 × 10^3^ HUVECs were seeded into each well of a 96‐well plate. Following adhesion, the HUVECs were cocultured with the macrophages under hypoxic conditions for 24 h. Subsequently, 10 μL of CCK‐8 reagent (Beyotime, China) was added to each well, and the hypoxic culture was continued. The absorbance of each well was then measured at 450 nm using a microplate reader (Thermo Fisher, USA). Each experiment was performed at least three times.

### 2.11 Tube Formation Assay

A tube formation assay was conducted to assess the ability of HUVECs to form tubule‐like structures. Briefly, 1 × 10^4^ HUVECs were seeded into each well of a 96‐well plate precoated with 50 μL of Matrigel (Corning, USA). Following 24 h of incubation, the formation of tubule structures was visualized using an inverted phase‐contrast microscope (Olympus, Japan). Images were analyzed with ImageJ software (National Institutes of Health, USA); the degree of tubule formation was quantified by measuring the total tubule length, number of branches, and number of nodes across five randomly selected fields of view (×100). Each experiment was performed at least three times.

### 2.12 Scratch Wound Healing Assay

A scratch wound healing assay was conducted to assess the migratory capacity of HUVECs. First, the conventional cell culture medium was replaced with serum‐free medium. A 200‐μL micropipette tip was subsequently used to create uniform scratches on the confluent HUVEC monolayer, and the cells were gently rinsed with PBS. After 24 h of incubation, the extent of wound closure was visualized using a microscope. The width of the wounds was measured with ImageJ software, and the migratory ability of the HUVECs was quantified based on the percentage of wound closure. Each experiment was performed at least three times.

### 2.13 Transwell Migration Assay

A Transwell migration assay was conducted to assess the migratory activity of HUVECs. Specifically, 1 × 10^4^ HUVECs were resuspended in serum‐free medium and seeded into the upper compartment of the Transwell chamber, whereas complete medium was added to the lower compartment to serve as a chemoattractant. After 24 h of incubation, the HUVECs that had migrated to the lower compartment were stained with crystal violet solution (Beyotime, China) and visualized under a light microscope. The number of stained migratory cells in the lower compartment was counted using ImageJ software, and this count was used to determine the migratory capacity of the HUVECs. Each experiment was performed at least three times.

### 2.14 Enzyme‐Linked Immunosorbent Assay (ELISA)

The concentration of ANGPT2 in cell culture supernatants was measured using a Human ANGPT2 ELISA Kit (Proteintech, China) following the manufacturer's instructions. Briefly, after extensive washing, biotinylated anti‐ANGPT2 detection antibodies were added and incubated at 37°C for 30 min. Following additional washes, HRP‐conjugated streptavidin working solution was added. The chromogenic reaction was initiated with tetramethylbenzidine (TMB) substrate and incubated in the dark at room temperature for 15 min. The reaction was stopped using a stop solution, and absorbance was measured at 450 nm with a microplate reader. Each experiment was performed at least three times.

### 2.15 Animals

Adult male Sprague–Dawley (SD) rats (260–280 g, a total of 180, *n* = 12 per group, 6 for western blotting, 6 for immunofluorescence) were purchased from Guangdong Medical Laboratory Animal Center (Guangdong, China) and housed under specific pathogen‐free (SPF)‐grade conditions with the temperature maintained at 22°C ± 2°C and the humidity maintained at 55% ± 5% under a 12 h light/dark cycle. The animals were acclimated to the environment for 1 week prior to the beginning of the experiments.

### 2.16 Rat Model of Chronic Cerebral Ischaemia Plus Revascularization Surgery

A 2‐vessel occlusion (2VO) plus encephalomyosynangiosis (EMS) model was established to simulate chronic cerebral ischaemia combined with revascularization surgery in rats, and the specific procedures are described in detail below.

#### 2.16.1 2VO

**FIGURE 1:**
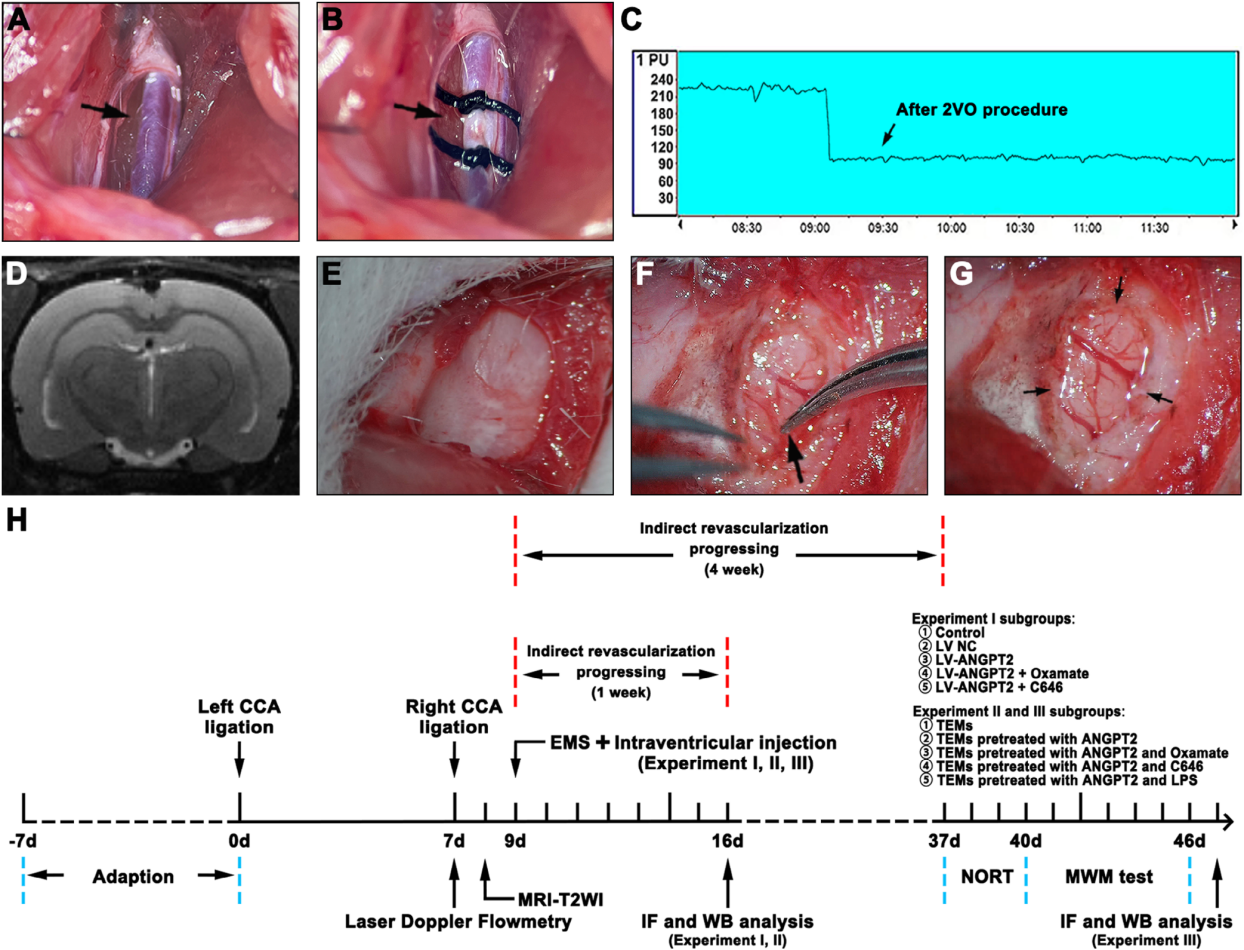
Procedures for establishing the 2VO + EMS model in SD rats. (A, B) Procedures for ligating the CCA (the black arrow indicates the left CCA). (C) Representative image of the analysis using the laser Doppler flow metre confirming the decrease in the CBF value during the 2VO procedure. (D) MRI‐T2WI showing no infarction after the 2VO procedure. Representative images showing the procedures of EMS surgery, including (E) the reflection of the skin and TM from the skull, (F, G) opening of the DM (the black arrow indicates the opened DM). (H) Experimental schedule. 2VO, 2‐vessel occlusion; CBF, cerebral blood flow; CCA, common carotid artery; DM, dura mater; EMS, encephalomyosynangiosis; MRI, magnetic resonance imaging; T2WI, T2‐weighted imaging; TM, temporal muscle.

**FIGURE 2:**
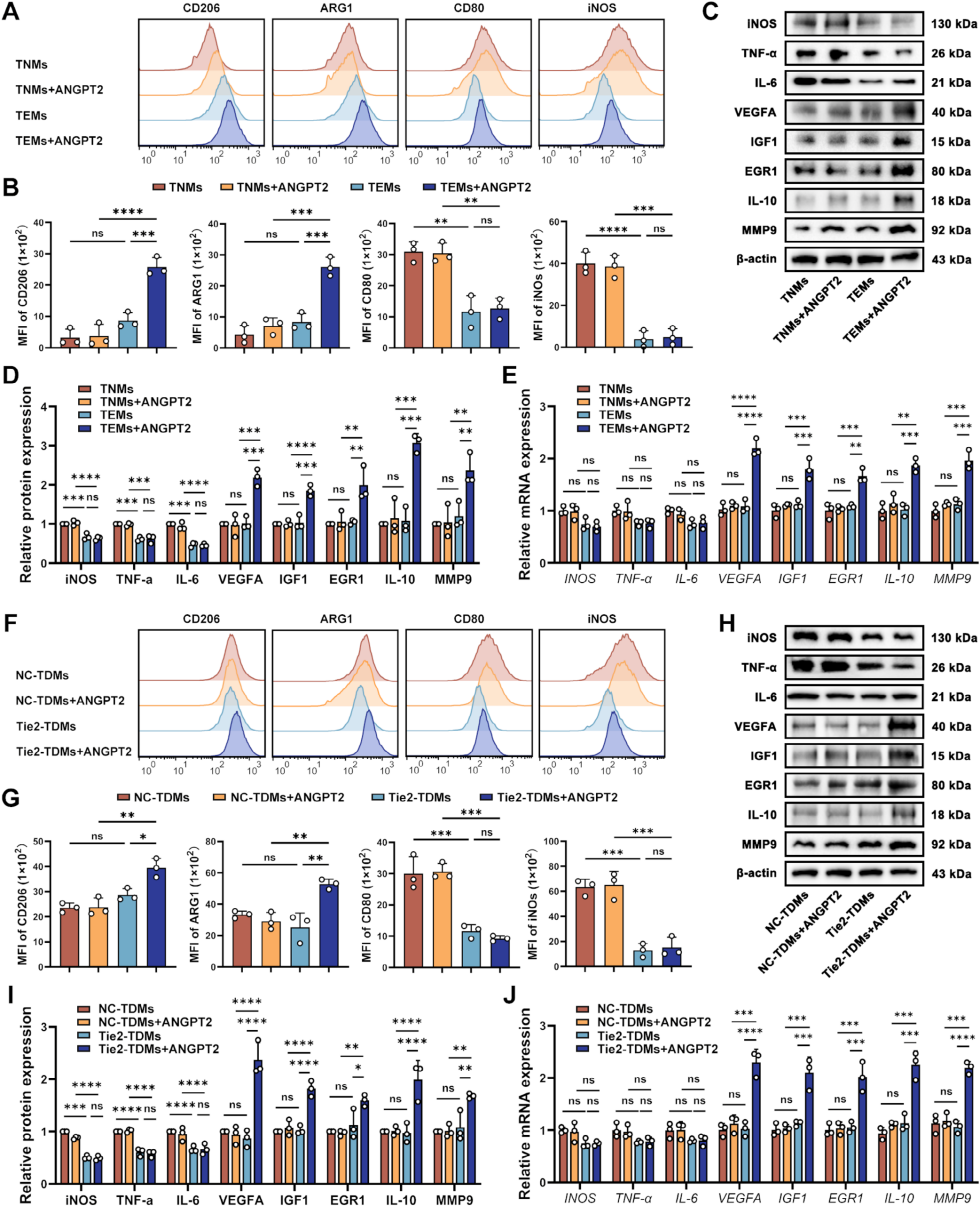
Results of flow cytometry, western blotting, and qPCR for determining the macrophage polarization status and the expression of proangiogenic factors. (A) Representative flow cytometry histogram showing the fluorescence intensity of 6 macrophage polarization markers. (B) Bar charts showing the MFIs of macrophage polarization markers (*n* = 3; one‐way ANOVA and Tukey's multiple comparisons test). (C) Representative western blot showing the relative levels of iNOS, TNF‐α, IL‐6, VEGFA, IGF1, EGR1, IL‐10, and MMP9 (normalized to β‐Actin expression) in primary macrophages. (D) Densitometric analyses of the relative levels of iNOS, TNF‐α, IL‐6, VEGFA, IGF1, EGR1, IL‐10, and MMP9 (*n* = 3; one‐way ANOVA and Tukey's multiple comparisons test). (E) qPCR results showing the relative mRNA expression of *iNOS*, *TNF‐α*, *IL‐6*, *VEGFA*, *IGF1*, *EGR1*, *IL‐10*, and *MMP9* in primary macrophages (*n* = 3; one‐way ANOVA and Tukey's multiple comparisons test). (F) Representative flow cytometry histogram showing the fluorescence intensity of macrophage polarization markers. (G) Bar charts showing the MFIs of macrophage polarization markers (*n* = 3; one‐way ANOVA and Tukey's multiple comparisons test). (H) Representative western blot showing the relative levels of iNOS, TNF‐α, IL‐6, VEGFA, IGF1, EGR1, IL‐10, and MMP9 (normalized to β‐Actin expression) in TDMs. (I) Densitometric analyses of the relative levels of iNOS, TNF‐α, IL‐6, VEGFA, IGF1, EGR1, IL‐10, and MMP9 (*n* = 3; one‐way ANOVA and Tukey's multiple comparisons test). (J) qPCR results showing the relative mRNA expression of *iNOS*, *TNF‐α*, *IL‐6*, *VEGFA*, *IGF1*, *EGR1*, *IL‐10*, and *MMP9* in TDMs (*n* = 3; one‐way ANOVA and Tukey's multiple comparisons test). The error bars represent the ± SDs. *p < 0.05, **p < 0.01, ***p < 0.001, and ****p < 0.0001. MFI, mean fluorescence intensity; TDMs, THP‐1‐derived macrophages; TEMs, Tie2‐expressing monocytes/macrophages; TNMs, Tie2‐negative monocytes/macrophages.

The rats were anesthetized with isoflurane (3% for induction, 1.5% for maintenance; RWD, China) with an oxygen flow rate of 0.6 L/min. A midline incision was made in the neck of the rat, and the left common carotid artery (CCA) was dissected. Double ligation of the left CCA was performed using two 5‐0 silk sutures. Seven days later, the right CCA was ligated using the same procedure (Figure 1A,B). Successful 2VO model establishment was confirmed by the following parameters: (1) a postoperative CBF value less than 40% of the baseline value detected by a laser Doppler blood flow meter (Figure 1C) and (2) no large infarct foci on the T2WI sequence of the MRI conducted 1 day after the 2VO procedure (Figure 1D).

#### 2.16.2 EMS

After confirming the successful establishment of the 2VO model, EMS surgery was performed on the rats. An incision of the skin and temporal muscle (TM) was then made, and a piece of skull (4 mm diameter) was ground away from the temporoparietal region using an electric drill. The dura mater (DM) and arachnoid membrane were carefully opened using forceps and a 1 mL syringe needle. The TM and DM were sutured together, and finally, the skin was sutured (Figure 1E–G). The experimental schedule was shown in Figure 1H.

#### 2.16.3 Intracerebroventricular Injection

Lentivirus expressing ANGPT2 (LV‐ANGPT2) and its negative control (LV NC) were designed and packaged by GenePharma Co. Ltd. (Shanghai, China). TEMs were resuspended in serum‐free Roswell Park Memorial Institute (RPMI) 1640 medium. The rat was anesthetized and fixed in a stereotaxic instrument (RWD, China). According to the rat brain atlas, a burr hole was drilled in the skull, and a Hamilton microsyringe (Hamilton Company, USA) was implanted into the right lateral ventricle. The stereotaxic coordinates of the microsyringe tip relative to bregma were as follows: mediolateral (ML) = 1.5 mm, anteroposterior (AP) = 1 mm, dorsoventral (DV) = 3.5 mm. A 10 μL suspension of lentivirus (1 × 10^8^ TU/mL) or cells (1 × 10^6^ cells) was injected at a constant rate of 1 μL/min. The microsyringe was retained in place for 10 min to avoid backflow before being slowly withdrawn.

### 2.17 Behavioral Tests

Morris Water Maze test (MWM) and Novel object recognition test (NORT) were performed on rat at 4 weeks after modeling. Results were analyzed using SMART version 3.0 software.

#### 2.17.1 NORT

The test was performed in an open‐field arena (80 × 80 × 80 cm) under indirect illumination (25–50 lx) to minimize reflections and shadows. During habituation, rats freely explored the empty arena for 10 min. In the familiarization phase, two identical objects were placed centrally, and exploration was allowed for 5 min. In the test phase, one familiar object was replaced with a novel object, and rats explored for 5 min. Object exploration was defined as sniffing or touching an object within 1 cm with the nose. Between trials, the arena and objects were cleaned with water to reduce odor cues. Rats were introduced facing away from the objects to avoid placement bias. Recognition memory was assessed using a discrimination index calculated from exploration times of the novel versus familiar object during the test phase.

#### 2.17.2 MWM Test

The MWM test was conducted in a circular pool (180 cm diameter, 60 cm height) filled with water (22°C ± 2°C), with the water level maintained 20 cm below the pool rim. The pool was conceptually divided into four equal quadrants. A transparent acrylic escape platform (15 cm diameter) was submerged 1 cm below the water surface in the center of the second quadrant. The first 5 days were the learning period, and the rats were released from one of the four quadrants facing the wall in random order. The rats needed to find the underwater platform within 60 s, and the escape latency and swimming route were recorded. If a rat did not find the platform, it was guided to the platform, and its escape latency was recorded as 60 s. Each rat was allowed to stay on the escape platform for 10 s regardless of whether the platform was found. The sixth day was the test day; the platform was removed, the rats were placed in the opposite quadrant from the target quadrant, and the escape latency, time spent in the target quadrant, number of platform crossings, and swimming route were recorded.

### 2.18 Brain Tissue Collection

The rats were anesthetized with Ulatan (20%, 1000 mg/kg), and the abdominal and thoracic cavities were incised to expose the liver and heart. The right atrial appendage was cut open, and normal saline was continuously injected into the left ventricle until the liver turned white. The skull was removed, and the brain tissue was collected. The samples used for immunofluorescence staining were fixed with 4% paraformaldehyde (PFA; Beyotime, China), while the samples used for western blotting were stored at −80°C.

### 2.19 Western Blotting

Western blotting was performed to determine the relative expression of proteins. Total protein was extracted from THP‐1 cells and brain tissues (approximately 1 mm around the EMS surgical site) using radioimmunoprecipitation assay (RIPA) buffer (Beyotime, China). After ultrasonic crushing and high‐speed centrifugation at 4°C, protein concentrations were measured with a bicinchoninic acid (BCA) protein assay kit (Beyotime, China). Subsequently, 20 μg of protein was separated on a 10% SDS–PAGE gel and transferred onto polyvinylidene fluoride (PVDF) membranes (Millipore, USA) with a 0.45 μm pore size. After an incubation with blocking buffer (Servicebio, China), the membranes were incubated with primary antibodies against iNOS (1:1000, 22226‐1‐AP, Proteintech, China), TNF‐α (1:1000, 17590‐1‐AP, Proteintech, China), IL‐6 (1:1000, ab233706, Abcam, UK), VEGFA (1:1000, 19003‐1‐AP, Proteintech, China), IGF1 (1:1000, ab106836, Abcam, UK), EGR1 (1:1000, 55117‐1‐AP, Proteintech, China), IL‐10 (1:1000, ab133575, Abcam, UK), MMP9 (1:1000, 10375‐2‐AP, Proteintech, China), Pan‐Kac (1:1000, PTM‐105RM, PTM BIO, China), pan‐Kla (1:1000, PTM‐1401RM, PTM BIO, China), H3K9la (1:1000, PTM‐1419RM, PTM BIO, China), H3K14la (1:1000, PTM‐1414RM, PTM BIO, China), H3K18la (1:1000, PTM‐1427RM, PTM BIO, China), H3K23la (1:1000, PTM‐1413RM, PTM BIO, China), H3K27la (1:1000, PTM‐1428RM, PTM BIO, China), H3K56la (1:1000, PTM‐1421RM, PTM BIO, China), H4K8la (1:1000, PTM‐1415RM, PTM BIO, China), H4K12la (1:1000, PTM‐1411RM, PTM BIO, China), LDHA (1:5000, 19987‐1‐AP, Proteintech, China), HK2 (1:20,000, 22029‐1‐AP, Proteintech, China), PKM2 (1:5000, 15822‐1‐AP, Proteintech, China), PFKFB3 (1:1000, 13763‐1‐AP, Proteintech, China), HDAC2 (1:10,000, 12922‐3‐AP, Proteintech, China), SIRT1 (1:3000, 13161‐1‐AP, Proteintech, China), p300 (1:500, 20695‐1‐AP, Proteintech, China), MCT4 (1:10,000, 22787‐1‐AP, Proteintech, China), Tie2 (1:2000, APRab18924, Enklife, China), phospho‐Tie2 (1:2000, APRab05559, Enklife, China), Tie2 (1:2000, APRab18924, Enklife, China), ANGPT2 (1:1000, 31499‐1‐AP, Proteintech, China), CD206 (1:1000, 18704‐1‐AP, Proteintech, China), ARG1 (1:1000, 16001‐1‐AP, Proteintech, China), CD31 (1:1000, 33075‐1‐AP, Proteintech, China), histone H3 (1:5000, 17168‐1‐AP, Proteintech, China), histone H4 (1:1000, 16047‐1‐AP, Proteintech, China), or β‐actin (1:20,000, 66009‐1‐Ig, Proteintech, China) in Tris‐buffered saline supplemented with 0.2% Tween‐20 (TBST) overnight at 4°C. After being washed, the membranes were incubated with HRP‐conjugated goat anti‐rabbit (1:10,000, ab6721, Abcam, UK) or HRP‐conjugated goat anti‐mouse (1:10,000, ab6728, Abcam, UK) antibodies at room temperature for 2 h. The PVDF membranes were subsequently imaged using a chemiluminescence system (Tanon, China). The images were analyzed using ImageJ software. Each experiment was repeated at least three times.

### 2.20 Immunofluorescence

Immunofluorescence was performed to determine the effects of ANGPT2/Tie2 signaling. The paraffin‐embedded sections of the brain tissue and PFA‐fixed cell slides were subjected to antigen retrieval with EDTA repair solution (Beyotime, China) in a boiling water bath for 30 min, permeabilized and blocked with 0.3% Triton X‐100 (Beyotime, China) and 5% BSA (Beyotime, China). Subsequently, the slices were incubated with primary antibodies against H3K18la (1:100, PTM‐1427RM, PTM BIO, China), CD206 (1:250, sc‐376232, Santa Cruz, USA), Tie2 (1:250, DF7500, Affinity, USA), Ki67 (1:50, AMM80747, Enklife, China), and CD31 (1:100, 33075‐1‐AP, Proteintech, China) in PBS supplemented with 0.2% Tween‐20 (PBST) overnight at 4°C. After washes, the slices were incubated with Alexa Fluor 488‐conjugated goat anti‐rabbit (1:2000, ab150077, Abcam, UK) and Alexa Fluor 594‐conjugated goat anti‐mouse (1:2000, ab150116, Abcam, UK) secondary antibodies at room temperature in the dark for 2 h. An Alexa Fluor 647‐conjugated anti‐CD11b antibody (0.5 μg, CL647‐65229, Proteintech, China) was used to directly stain CD11b. The slices were sealed with mounting medium supplemented with DAPI (Abcam, UK), and fluorescence images were acquired using a confocal microscope (Olympus, Japan).

### 2.21 Statistical Analysis

Statistical analyses were performed using the Statistical Program for Social Science (SPSS) version 23.0 (IBM Corporation, USA), alongside GraphPad Prism version 10 (GraphPad Software, USA). The statistical approaches applied included one‐way analysis of variance (ANOVA) or two‐way ANOVA. Experimental data from repeated trials are presented as the means ±SDs. Statistically significant differences were indicated when *p* < 0.05.

## 3 Results

### 3.1 ANGPT2/Tie2 Signaling Induced M2 Macrophage Polarization and Increased the Expression of Proangiogenic Factors

We isolated primary TEMs and treated them with exogenous ANGPT2 to investigate the effect of ANGPT2 on Tie2 signaling in macrophages. The direct binding of ANGPT2 to Tie2 was confirmed by co‐immunoprecipitation (Figure S3). Following ANGPT2 exposure, the flow cytometry analysis revealed increased expression of the M2 macrophage markers CD206 and ARG1 but no significant change in the expression of the M1 macrophage markers CD80 and iNOS (Figure 2A,B). Moreover, WB analysis and qPCR showed that the expression of representative M2‐related genes and proangiogenic factors (VEGFA, IGF1, EGR1, IL‐10, and MMP9) was significantly increased, while no differences were observed in the expression of M1‐related genes (iNOS, TNF‐α, and IL‐6) (Figure 2C–E).

We constructed Tie2‐overexpressing TDMs (Tie2‐TDMs) to ensure the reproducibility of the experiment. Similarly, exogenous ANGPT2 significantly drove Tie2‐TDMs towards the M2 phenotype, accompanied by increased expression of VEGFA, IGF1, EGR1, IL‐10, and MMP9 (Figure 2F–J). The subsequent experiments performed in this study utilized Tie2‐TDMs as surrogates for primary TEMs.

### 3.2 ANGPT2/Tie2 Signaling Promoted Macrophage M2 Polarization by Upregulating LDHA and Subsequently Increasing H3K18la

**FIGURE 3:**
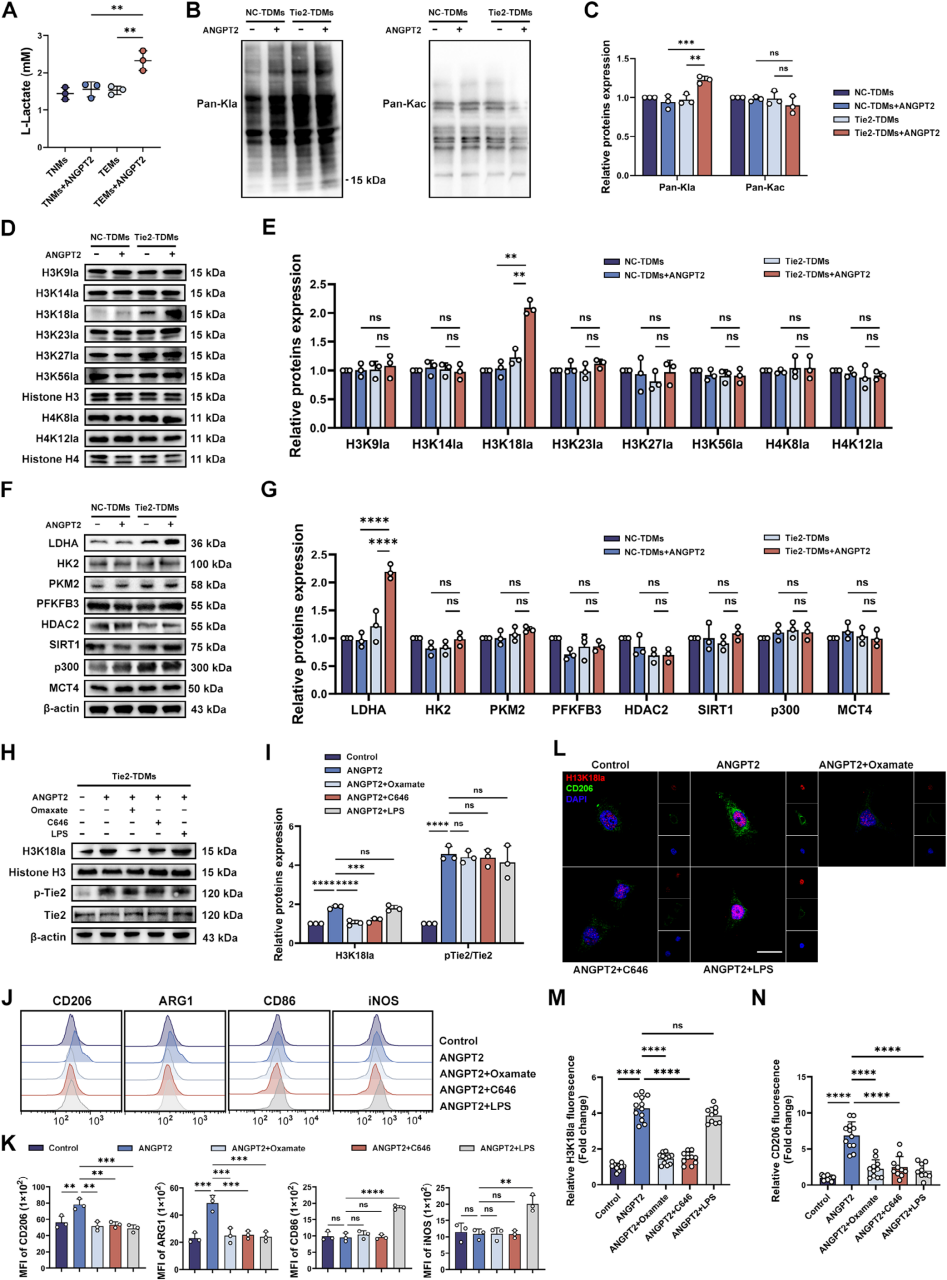
Western blot, immunofluorescence, and flow cytometry results showing Kla levels and the macrophage polarization status. (A) Scatter plot showing the intracellular l‐lactate concentration in TDMs from each group. (B) Representative western blot showing the relative levels of Kla and Kac (normalized to β‐Actin expression) in the TDMs from each group. (C) Densitometric analyses of the relative levels of pan‐Kla and pan‐Kac (*n* = 3; one‐way ANOVA and Tukey's multiple comparisons test). (D) Representative western blot showing the relative levels of H3K9la, H3K14la, H3K18la, H3K23la, H3K27la, H3K56la (normalized to H3 expression), H4K8la, and H4K12la (normalized to H4 expression) in the TDMs from each group. (E) Densitometric analyses of the relative levels of H3K9la, H3K14la, H3K18la, H3K23la, H3K27la, H3K56la, H4K8la, and H4K12la (*n* = 3; one‐way ANOVA and Tukey's multiple comparisons test). (F) Representative western blot showing the relative levels of LDHA, HK2, PKM2, PFKFB3, HDAC2, SIRT1, p300, and MCT4 (normalized to β‐Actin expression) in the TDMs from each group. (G) Densitometric analyses of the relative levels of PFKFB3, HDAC2, SIRT1, p300, and MCT4 (*n* = 3; one‐way ANOVA and Tukey's multiple comparisons test). (H) Representative western blot showing the relative levels of H3K18la (normalized to H3 expression), pTie2 and Tie2 (normalized to β‐Actin expression). (I) Densitometric analyses of the relative levels of H3K18la and pTie2/Tie2 (*n* = 3; one‐way ANOVA and Tukey's multiple comparisons test). (J) Representative flow cytometry histogram showing the fluorescence intensity of macrophage polarization markers. (K) Bar charts showing the MFIs of macrophage polarization markers (*n* = 3; one‐way ANOVA and Tukey's multiple comparisons test). (L) Representative images of immunofluorescence staining showing the expression of H3K18la and CD206 in the TDMs of each group. (M, N) Bar charts showing the fluorescence intensity of each cell (*n* = 9–12; one‐way ANOVA and Tukey's multiple comparisons test). The error bars represent the ± SDs. *p < 0.05, **p < 0.01, ***p < 0.001, and ****p < 0.0001. MFI, mean fluorescence intensity; TDMs, THP‐1‐derived macrophages.

We detected the l‐lactate concentration and Kla levels to clarify the specific mechanism by which ANGPT2/Tie2 regulates the M2 polarization of macrophages. After ANGPT2 treatment, the l‐lactate concentration was significantly increased in Tie2‐TDMs, which was accompanied by a concurrent increase in the pan‐Kla levels, while pan‐Kac level showed no difference (Figure 3A–C). Given the critical role of histone lactylation in macrophage polarization and the notable difference observed in the 10–15 kDa band using the pan‐Kla antibody, we further examined the lactylation status of histone H3/H4 and observed increases in H3K18la (Figure 3D,E). Next, we analyzed the expression of key proteins involved in lactate metabolism and lactation modification, including lactate‐metabolizing enzymes (LDHA, HK2, PKM2, PFKFB3), lactylation erasers (HDAC2, SIRT1), lactylation writer p300, and lactate efflux protein MCT4. Results showed that ANGPT2 treatment significantly increased LDHA levels in Tie2‐TDMs (Figure 3F,G).

Furthermore, we utilized Oxamate (a specific LDHA inhibitor, 10 mM) and C646 (a specific p300 histone acetyltransferase inhibitor, 10 μM) to reduce histone lactylation levels and lipopolysaccharide (LPS, an M1 polarization inducer) to induce M1 macrophage polarization [24, 25, 26]. Oxamate and C646 treatment significantly downregulated H3K18la levels and decreased M2 polarization, whereas LPS exclusively promoted M1 polarization but did not affect H3K18la levels. No significant difference in Tie2 phosphorylation levels was detected between the ANGPT2‐treated groups, indicating that both histone lactylation and subsequent macrophage polarization are downstream processes of the ANGPT2/Tie2 pathway (Figure 3H–K). Immunofluorescence results visually showed that ANGPT2 upregulated the expression of H3K18la and CD206 (a marker of M2 macrophage), while Oxamate and C646 reduced CD206 expression by decreasing H3K18la, and LPS only decreased CD206 expression without significant effects on H3K18la (Figure 3L–N).

### 3.3 ANGPT2/Tie2 Signaling Increased the Binding of H3K18la to the Promoters of M2‐Related Genes and Proangiogenic Factors

**FIGURE 4:**
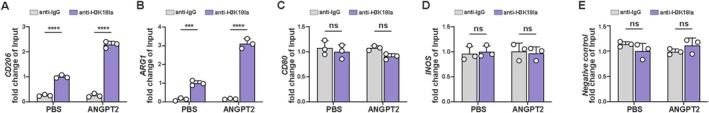
Results of ChIP–qPCR assays verifying the regulatory effect of ANGPT2/Tie2 signaling on H3K18la‐mediated transcriptional regulation. (A–E) Bar charts showing the relative expression of *CD206*, *ARG1*, *CD80*, *INOS*, and negative control (noncoding sequences). The error bars represent the ± SDs. *p < 0.05, **p < 0.01, ***p < 0.001, and ****p < 0.0001.

We performed ChIP–qPCR assays to further verify the regulatory effect of ANGPT2/Tie2 on H3K18la‐mediated transcriptional regulation. The results showed that the precipitates using the H3K18la‐specific antibody significantly enriched the promoter regions of M2 macrophage marker genes (*CD206* and *ARG1*). Notably, pretreatment with ANGPT2 further increased H3K18la enrichment at the promoters of the aforementioned M2‐related genes (Figure 4). In contrast, no significant differences in qPCR signals for M1 macrophage marker genes (*CD80* and *INOS*) were detected between the H3K18la antibody and IgG antibody, regardless of ANGPT2 treatment. These findings provide direct evidence that ANGPT2/Tie2 signaling promotes H3K18la enrichment at the promoters of M2‐associated genes, potentially facilitating their transcriptional activation and shifting macrophages towards a tissue repair and proangiogenic phenotype.

### 3.4 ANGPT2/Tie2 Signaling Induces H3K18la‐Mediated M2 Polarization of Macrophages to Promote HUVEC Proliferation

**FIGURE 5:**
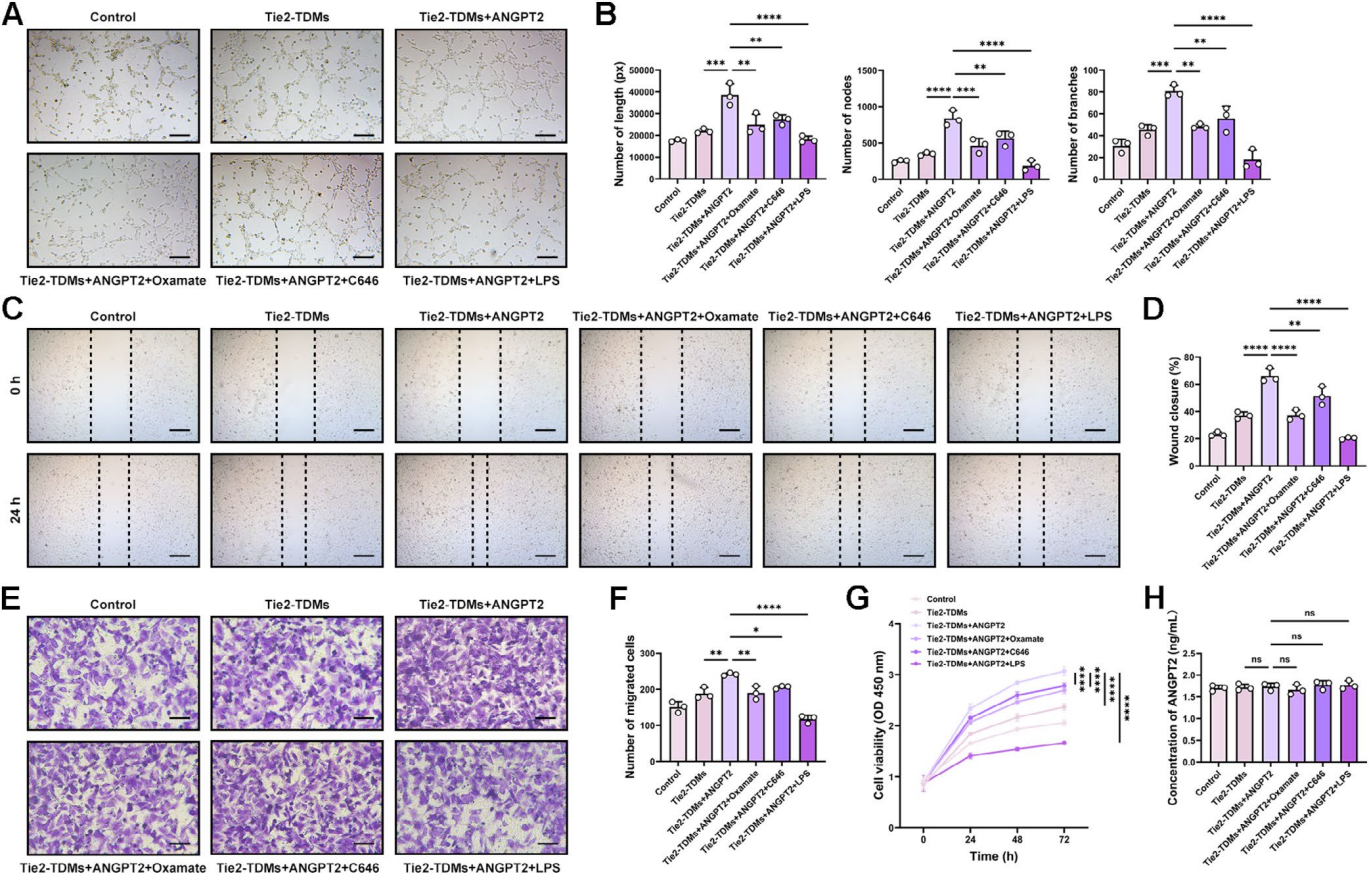
Results of functional experiments reflecting the proliferative ability and viability of HUVECs. (A) Representative images showing the results of the tube formation assays for each group. Bar = 200 μm. (B) The total length, number of branches, and number of nodes were determined (*n* = 3; one‐way ANOVA and Tukey's multiple comparisons test). (C) Representative images showing the results of the scratch wound healing assays at 24 h for each group. Bar = 200 μm. (D) The results are presented as the percentage of wound closure at 0–24 h (*n* = 3; one‐way ANOVA and Tukey's multiple comparisons test). (E) Representative fields showing the results of the Transwell migration assays for each group. Bar = 100 μm. (F) The results were determined by counting the number of migrated cells per field (*n* = 3; one‐way ANOVA and Tukey's multiple comparisons test). (G) Line graph showing the results for cell viability evaluated using the CCK‐8 assay (*n* = 3; two‐way ANOVA and Tukey's multiple comparisons test). (H) ELISA results showing the concentrations of ANGPT2 in coculture supernatant (*n* = 3; one‐way ANOVA and Tukey's multiple comparisons test). HUVECs: Human umbilical vein endothelial cells. The error bars represent the ± SDs. *p < 0.05, **p < 0.01, ***p < 0.001, ****p < 0.0001. HUVECs, human umbilical vein endothelial cells.

A series of functional assays were conducted to evaluate the effect of ANGPT2/Tie2‐regulated macrophages on HUVECs. Tube formation assays revealed that ANGPT2‐pretreated Tie2‐TDMs markedly increased the number of tube branches and total tube length of HUVECs, reflecting increased angiogenic potential (Figure 5A,B, Figure S4A). In scratch wound healing assays, compared with the control group, HUVECs cocultured with ANGPT2‐pretreated Tie2‐TDMs displayed accelerated wound closure, with a significantly higher healing rate at 24 h (Figure 5C,D). Consistently, Transwell migration assays revealed a significant increase in the number of migrating HUVECs in the presence of ANGPT2‐pretreated Tie2‐TDMs (Figure 5E,F, Figure S4B). Furthermore, a CCK8 assay showed that HUVECs cocultured with ANGPT2‐pretreated Tie2‐TDMs exhibited significantly higher absorbance at 450 nm (Figure 5G), indicating increased proliferative activity. Pretreatment of Tie2‐TDMs with additional Oxamate, C646, or LPS significantly inhibited HUVEC activity. ELISA results showed no difference in ANGPT2 levels in the supernatant among the groups (Figure 5H). Taken together, these findings confirm that ANGPT2‐pretreated Tie2‐TDMs robustly increase the viability of HUVECs, including promoting proliferation, tube formation, and migration.

### 3.5 ANGPT2 Enhanced the H3K18la‐Mediated M2 Polarization of TEMs to Promote Angiogenesis in the CIB

**FIGURE 6:**
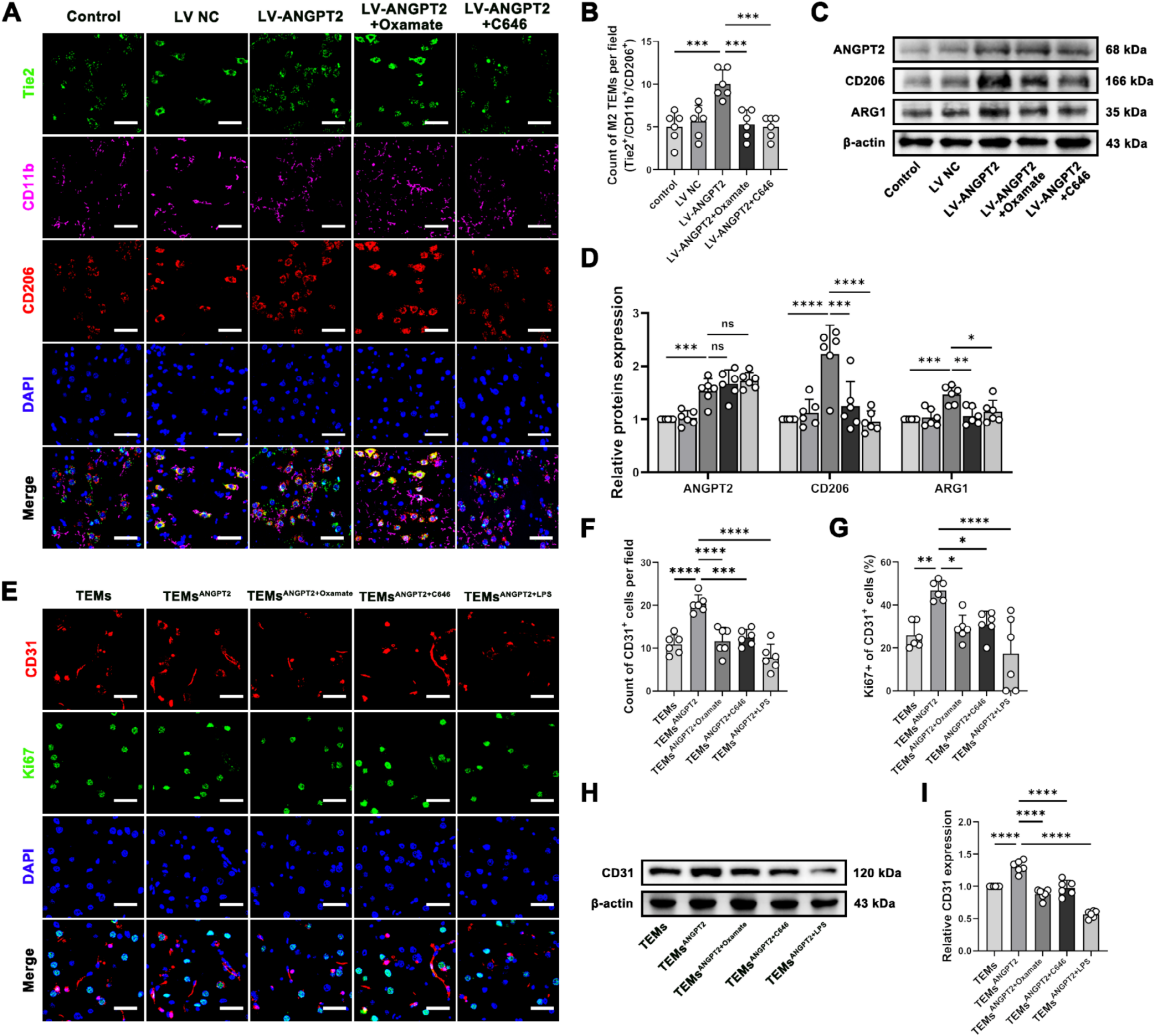
Western blot and immunofluorescence staining showing the expression of relevant cytokines in the CIB of 2VO + EMS rats. (A) Representative images of triple immunofluorescence staining showing Tie2^+^, CD11b^+^, and CD206^+^ cells in the CIB of each group. Bar = 50 μm. (B) Counts of M2 TEMs (Tie2^+^/CD11b^+^/CD206^+^) in each group (*n* = 6; one‐way ANOVA and Tukey's multiple comparisons test). (C) Representative western blot showing the relative expression of ANGPT2, CD206, and ARG1 (normalized to β‐Actin expression). (D) Densitometric analyses of the relative expression of ANGPT2, CD206, and ARG1 (*n* = 6; one‐way ANOVA and Tukey's multiple comparisons test). (E) Representative images of double immunofluorescence staining showing CD31^+^ and Ki67^+^ cells in the CIB of each group. Bar = 50 μm. (F) Column chart showing the counts of CD31^+^ cells in each group (*n* = 6; one‐way ANOVA and Tukey's multiple comparisons test). (G) Column chart showing the proportion of Ki67‐positive ECs in each group (*n* = 6; one‐way ANOVA and Tukey's multiple comparisons test). (H) Representative western blot showing the relative expression of CD31 (normalized to β‐Actin expression). (I) Densitometric analyses of the relative expression of CD31 (*n* = 6; one‐way ANOVA and Tukey's multiple comparisons test). (H) *p < 0.05, **p < 0.01, ***p < 0.001, and ****p < 0.0001. The error bars represent the ±SDs. CIB: Chronically ischaemic brain; TEMs, Tie2‐expressing monocytes/macrophages.

We established a 2VO + EMS rat model and injected LV‐ANGPT2 into the right lateral ventricle to confirm the aforementioned effects in vivo. Immunofluorescence staining showed that LV‐ANGPT2 injection into the right lateral ventricle resulted in increased M2‐type TEMs infiltration in the CIB, whereas the tail vein injection of Oxamate or C646 markedly decreased M2‐type TEMs infiltration (Figure 6A,B). WB results also showed that overexpressing ANGPT2 significantly increased the expression of CD206 and ARG1 in CIB (Figure 6C,D). Furthermore, we performed intracerebroventricular injections in rats using primary TEMs isolated from each rat's own blood. The results revealed that the intraventricular injection of ANGPT2‐pretreated TEMs significantly increased the expression of CD31 (a marker of ECs) and the proportion of Ki67‐positive ECs in the CIB, whereas the intraventricular injection of ANGPT2/Oxamate‐pretreated, ANGPT2/C646‐pretreated, or ANGPT2/LPS‐pretreated TEMs abrogated these increases (Figure 6E–I).

**FIGURE 7:**
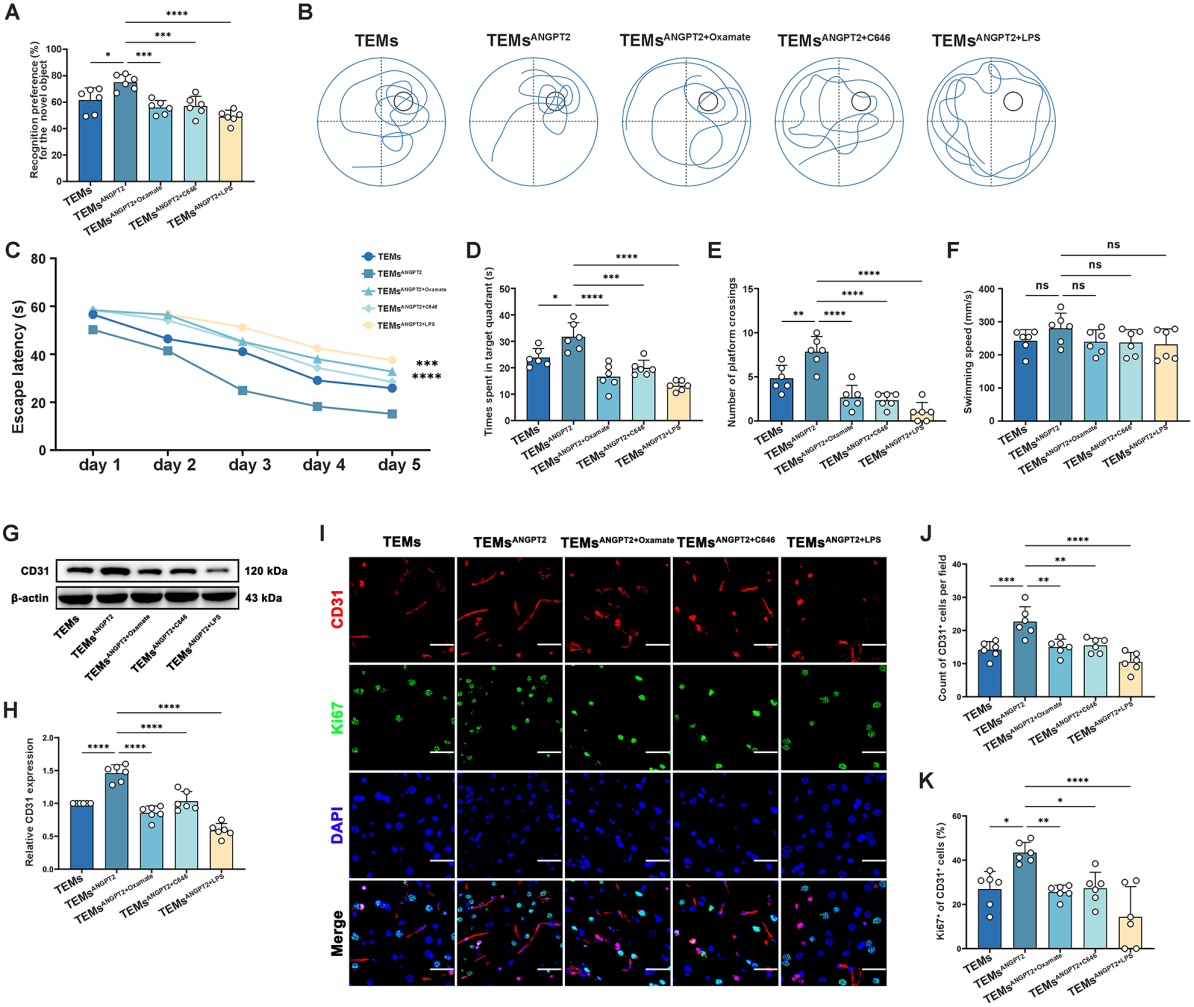
Behavioral test, western blotting, and immunofluorescence results in 2VO + EMS rats 4‐week post‐modeling. (A) Quantitative analysis of the percentage of recognition preference for the novel object in each group rats in NORT test (*n* = 6; one‐way ANOVA and Tukey's multiple comparisons test). (B) Representative image showing the swimming paths in each group rats during the MWM test. (C) Line chart showing the average escape latencies in each group rats during the MWM test (*n* = 6; two‐way ANOVA and Tukey's multiple comparisons test; ***: TEMs vs. TEMs^ANGPT2^, ****: TEMs^ANGPT2^ vs. TEMs^ANGPT2+Oxamate^, TEMs^ANGPT2^ vs. TEMs^ANGPT2+C646^, TEMs^ANGPT2^ vs. TEMs^ANGPT2+LPS^). (D) Quantitative analysis of the time spent in target quadrant in each group rats during the MWM test (*n* = 6; one‐way ANOVA and Tukey's multiple comparisons test). (E) Quantitative analysis of the number of platform crossings in each group rats during the MWM test (*n* = 6; one‐way ANOVA and Tukey's multiple comparisons test). (F) Quantitative analysis of the average swimming speed in each group rats during the MWM test (*n* = 6; one‐way ANOVA and Tukey's multiple comparisons test). (G) Representative western blot showing the relative expression of CD31 (normalized to β‐Actin expression). (H) Densitometric analyses of the relative expression of CD31 (*n* = 6; one‐way ANOVA and Tukey's multiple comparisons test). (I) Representative images of double immunofluorescence staining showing CD31^+^ and Ki67^+^ cells in the CIB of each group. Bar = 50 μm. (J) Column chart showing the counts of CD31^+^ cells in each group (*n* = 6; one‐way ANOVA and Tukey's multiple comparisons test). (K) Column chart showing the proportion of Ki67‐positive ECs in each group (*n* = 6; one‐way ANOVA and Tukey's multiple comparisons test). The error bars represent the ± SDs. *p < 0.05, **p < 0.01, ***p < 0.001, ****p < 0.0001. 2VO, 2‐vessel occlusion; EMS, encephalomyosynangiosis; MWM, Morris water maze; NORT, novel object recognition test; TEMs, Tie2‐expressing monocytes/macrophages.

Behavioral experiments after 1 week of modeling did not reveal any differences (Figure S5). Four weeks after modeling, the NORT results revealed that the rats injected with ANGPT2‐pretreated TEMs recognized the novel object significantly more often than other groups (Figure 7A). In the MWM tests, the rats injected with ANGPT2‐pretreated TEMs had significantly shorter escape latencies on Day 5, longer durations in the target quadrant, and greater numbers of platform crossings on the test day. However, there was no significant difference in swimming speed among the groups (Figure 7B–F). Similar to the tests one week after modeling, intraventricular injection of ANGPT2‐pretreated TEMs significantly increased the expression of CD31 and the proportion of Ki67‐positive ECs in the CIB (Figure 7G–K).

## 4 Discussion

The specific mechanisms by which TEMs promote EC proliferation remain unclear. Here, we identified that ANGPT2 has more functions than simply recruiting TEMs; it also induces H3K18 lactylation by upregulating LDHA, thereby promoting M2 macrophage polarization. Guided by this insight, this study revealed that ANGPT2/Tie2 promotes EC proliferation and angiogenesis in the CIB by inducing H3K18la‐mediated M2 macrophage polarization. Detailed discussions are presented below.

Tie2 was previously identified as an EC‐specific receptor for ANGPTs, with ANGPT1 and ANGPT2 acting as its competitive ligands [7]. ANGPT1 binding induces Tie2 phosphorylation, which in turn enhances vascular maturation and stability. By contrast, ANGPT2 binding inhibits Tie2 phosphorylation, thereby triggering vascular destabilization and facilitating angiogenesis [4]. TEMs, a cell subset first identified in breast cancer by De Palma's group, have been shown to promote angiogenesis across multiple disease models [27]. Furthermore, accumulating evidence has established that ANGPT2, rather than its homolog ANGPT1, serves as the pivotal factor orchestrating the functional phenotype and biological behavior of TEMs. Notably, TEMs have recently been shown to be actively recruited to the CIB by ANGPT2, where they potently stimulate EC proliferation and angiogenesis [27]. Macrophage function is tightly linked to polarization plasticity, with M1/M2 polarization representing a key functional dichotomy: the classically activated M1 phenotype drives inflammation and antigen presentation, whereas the alternatively activated M2 phenotype orchestrates tissue repair, angiogenesis, and immunoregulation [28, 29]. Within the tumor microenvironment, TEMs are categorized as a specialized type of TAM [30]. Owing to their robust secretion of proangiogenic factors, they have been described as more M2‐like TAMs [14]. The specific expression of Tie2 results in unique functional properties of TEMs that distinguish them from conventional monocytes/macrophages; however, the underlying regulatory mechanism remains elusive.

We investigated this process by treating primary TEMs with exogenous ANGPT2 and observed a significant increase in M2 polarization. Concomitantly, the expression of proangiogenic factors was markedly upregulated. Interestingly, although ANGPT2 pretreatment did not affect the M1 polarization of either TEMs or TNMs, we observed that the level of M1 polarization of TEMs was lower than that of TNMs, regardless of whether exogenous ANGPT2 was applied. This phenomenon may be attributed to the basal activity of Tie2, which exerts a mild inhibitory effect on M1 macrophage polarization.

Given the poor in vitro proliferative capacity of primary monocytes/macrophages, coupled with the distinct biological effects that cells from different individuals may exhibit due to patients' genetic variability, these factors pose certain challenges to the stability and reproducibility of the experiments. Therefore, we generated Tie2‐overexpressing THP‐1 cells and induced their differentiation into macrophages (i.e., Tie2‐TDMs). ANGPT2 treatment also promoted M2 polarization and increased the expression of proangiogenic factors in Tie2‐TDMs, a trend consistent with that observed in primary TEMs. Consequently, the subsequent experiments performed in this study utilized Tie2‐TDMs as surrogates for primary TEMs.

A hallmark of the tumor microenvironment is the accumulation of lactate, a byproduct of aerobic glycolysis (Warburg effect) in cancer cells, which creates an acidic niche [31, 32]. Chronic hypoxia also induces upregulation of glycolysis, leading to increased lactate levels [33, 34]. Recent studies have identified histone lactylation as a novel epigenetic modification that connects the metabolic status and gene expression: lactate, once considered a mere metabolic waste, acts as a substrate to covalently modify lysine residues on histones, thereby altering the accessibility of chromatin and regulating the transcription of target genes [15, 35]. Specifically, histone lactylation participates in regulating the expression of M2‐associated genes (e.g., *CD206* and *ARG1*), thereby regulating immune cell functions and promoting tissue repair and homeostasis [21, 22, 23]. A study by Chen's group revealed that during macrophage polarization, changes in the histone lactylation modification system predominate when macrophages shift to the M2 phenotype, among which the change in H3K18la levels is the most significant [36].

Based on the aforementioned findings, we hypothesized that ANGPT2/Tie2 signaling modulates macrophage polarization by regulating histone lactylation levels. We tested this hypothesis by detecting L‐lactate concentrations and Kla levels and confirmed that ANGPT2/Tie2 signaling indeed upregulated H3K18la levels. Furthermore, the expression of key proteins implicated in lactate metabolism and lactylation modification was evaluated, with a marked upregulation of LDHA observed in the ANGPT2 treatment group, suggesting the mechanism by which ANGPT2/Tie2 induces the increase of H3K18la. We subsequently used Oxamate (a specific LDHA inhibitor), C646 (a specific p300 histone acetyltransferase inhibitor), and LPS (an M1 polarization inducer) to reduce lactate production and induce M1 polarization, respectively. The results revealed that upon the Oxamate or C646‐mediated reduction in H3K18la levels, M2 polarization was concomitantly decreased; in contrast, the LPS‐induced M1 polarization of macrophages failed to decrease H3K18la. Oxamate reduces the substrate availability for histone lactylation by decreasing lactate production; C646 targets the catalytic activity of p300, thereby blocking the modification process of histone lactylation. The individual application of Oxamate and C646 further highlighted the core role of H3K18la in regulating downstream biological phenotypes. These findings strongly indicated that H3K18la acts as an upstream regulator of M2 polarization, which is consistent with the findings of previous studies [37]. Subsequent ChIP–qPCR assays further confirmed that H3K18la is enriched at the promoters of M2‐associated genes, with this enrichment being further increased following the activation of ANGPT2/Tie2 signaling. These results not only aligned with ChIP‐seq findings from other studies but also revealed, at the molecular level, the regulatory mechanism of the ANGPT2/Tie2 axis in macrophage polarization [38, 39].

M2 macrophages typically play critical roles in tissue repair and proangiogenic processes [40, 41]. We cocultured ANGPT2‐pretreated Tie2‐TDMs with HUVECs to further validate the regulatory role of macrophage ANGPT2/Tie2 signaling in EC proliferation, and the results showed that under these conditions, the proangiogenic activity of Tie2‐TDMs was markedly increased. Furthermore, the results of in vivo experiments demonstrated that the ANGPT2 overexpression led to a significant increase in the number of M2‐type TEMs infiltrating the CIB in a 2VO rat model of chronic cerebral ischaemia. In contrast, the tail vein injection of Oxamate or C646 resulted in a less M2‐type TEMs infiltration, providing preliminary evidence that Oxamate and C646 inhibits the M2 polarization of macrophages induced by the ANGPT2/Tie2 pathway in vivo. However, since Oxamate and C646 are also taken up by ECs, their potential to reduce lactate production in ECs might interfere with the regulation of EC proliferation; thus, we planned to directly inject ANGPT2‐pretreated TEMs. Considering that the direct injection of TEMs may cause immune rejection, we extracted each rat's own TEMs for intracerebroventricular injection. The results showed that ANGPT2‐pretreated TEMs markedly promoted EC proliferation in the CIB, ultimately improving cognitive function in rats. The negative MWM test results at 1 week post‐modeling are likely due to the relatively short time after EMS surgery, resulting in limited new blood vessels, which were not yet sufficient to significantly improve the cognitive function of rats. The negative result at this time point is also consistent with our previous research conclusions; significant recovery of cognitive function usually occurs 3–5 weeks post EMS surgery [42]. Collectively, these in vitro and in vivo findings confirm that the ANGPT2‐induced M2 polarization of TEMs is a key contributor to promoting EC proliferation and angiogenesis in the CIB, thereby providing direct experimental evidence to support the ANGPT2/Tie2–H3K18la–M2 polarization regulatory axis proposed in this study.

This study has several limitations. First, the specific mechanism by which ANGPT2/Tie2 increases H3K18la awaits full elucidation, even though we have identified LDHA as a potential link between this pathway and H3K18la modification. Second, the protocol of inhibiting histone lactylation employed in our experiments lacks sufficient specificity, and future investigations could adopt more targeted strategies such as CRISPR/Cas9‐mediated H3K18 site mutations to further validate the functional role of H3K18la. Third, whether the increase in H3K18la levels is regulated by exogenous lactate remains unclear. Fourth, in our animal experiments, we cannot fully exclude the possibility that ANGPT2 regulates Tie2 expressed on ECs, nor can we rule out the influence of endogenous ANGPT2 on TEMs, even though our two animal studies (which involved injections of LV‐ANGPT2 and primary TEMs) support the present conclusion.

## 5 Conclusions

ANGPT2/Tie2 signaling upregulates LDHA and increases H3K18la, thereby driving macrophage M2 polarization. These M2‐polarized macrophages secrete high levels of proangiogenic factors, which in turn exert stimulatory effects on EC proliferation and angiogenesis in the CIB.

## Author Contributions

**Chuyang Tai:** writing – original draft, formal analysis, methodology, investigation. **Cong Ling:** writing – original draft, formal analysis, methodology, investigation. **Yang Yang:** writing – original draft, formal analysis, methodology, investigation. **Ni Mo:** writing – review and editing, data curation, investigation. **Cian Yao:** writing – review and editing, visualization, investigation. **Songtian Lv:** writing – review and editing, visualization, investigation. **Baoyu Zhang:** writing – review and editing, investigation. **Hui Wang:** writing – review and editing, conceptualization, supervision. **Chuan Chen:** writing – review and editing, conceptualization, supervision, funding acquisition. All authors read and approved the final manuscript.

## Funding

This research was supported by the Guangdong Basic and Applied Basic Research Foundation under Grant No. 2025A1515012390 and Grant No. 2026A1515012992; the Basic Research Project of Sun Yat‐sen University under Grant No. b202408011053050001; and the Guangzhou Key Research and Development Program under Grant No. 2023B03J0191.

## Conflicts of Interest

The authors declare no conflicts of interest.

## Supporting information


**Figure S1:** Identification of primary TEMs and TNMs. (A) Representative flow cytometric dot plot identifying Tie2^+^ monocytes (Tie2^+^/CD14^+^) and Tie2^−^ monocytes (Tie2^−^/CD14^+^). (B) Representative immunofluorescence images identifying Tie2^+^ monocytes (Tie2^+^/CD14^+^) and Tie2^−^ monocytes (Tie2^−^/CD14^+^). Bar = 20 μm. (C) Induction of monocytes to differentiate into macrophages. Bar = 200 μm. TEMs, Tie2‐expressing monocytes/macrophages; TNMs, Tie2‐negative monocytes/macrophages.
**Figure S2:** Preparation of TDMs. (A) Bright‐field and fluorescence images showing the morphology of THP‐1 cells after transfection. Bar = 200 μm. (B) Column chart showing the expression level of Tie2 mRNA in THP‐1 cells transfected with LV‐Tie2 and its negative control (*n* = 3, unpaired *t*‐test). (C) Plasmid structure provided by the manufacturer (ReGene Biotechnology Co. Ltd., Guangzhou, China). (D) Induction of THP‐1 cells to differentiate into TDMs. Bar = 200 μm. TDMs, THP‐1‐derived macrophages.
**Figure S3:** Co‐Immunoprecipitation assays verifying the direct binding of ANGPT2 to Tie2.
**Figure S4:** Images of tube formation assay and Transwell migration assay processed with ImageJ.
**Figure S5:** MWM test results in 2VO + EMS rats 1 week post‐modeling. (A) Representative image showing the swimming paths in each group rats during the MWM test. (B) Line chart showing the average escape latencies in each group rats during the MWM test. (C) Quantitative analysis of the time spent in target quadrant in each group rats during the MWM test. (D) Quantitative analysis of the number of platform crossings in each group rats during the MWM test. (E) Quantitative analysis of the average swimming speed in each group rats during the MWM test. MWM: Morris water maze.
**Table S1:** Primers used in qPCR in this study.
**Table S2:** Primers used in ChIP‐qPCR in this study.

## Data Availability

The data that support the findings of this study are available from the corresponding author upon reasonable request.
