# Supplementary material for: ANGPT2/Tie2 Enhances H3K18la‐Mediated Macrophage M2 Polarization to Promote Endothelial Cell Proliferation in the Chronically Ischaemic Brain
Source: CNS Neurosci Ther. 2026 Apr 12;32(4):e70879. doi: 10.1002/cns.70879 (PMC13070873; doi:10.1002/cns.70879)
Supplement: Supplementary file 1 — Figure S1: Identification of primary TEMs and TNMs. (A) Representative flow cytometric dot plot identifying Tie2+ monocytes (Tie2+/CD14+) and Tie2− monocytes (Tie2−/CD14+). (B) Representative immunofluorescence images identifying Tie2+ monocytes (Tie2+/CD14+) and Tie2− monocytes (Tie2−/CD14+). Bar = 20 μm. (C) Induction of monocytes to differentiate into macrophages. Bar = 200 μm. TEMs, Tie2‐expressing monocytes/macrophages; TNMs, Tie2‐negative monocytes/macrophages. Figure S2: Preparation of TDMs. (A) Bright‐field and fluorescence images showing the morphology of THP‐1 cells after transfection. Bar = 200 μm. (B) Column chart showing the expression level of Tie2 mRNA in THP‐1 cells transfected with LV‐Tie2 and its negative control (n = 3, unpaired t‐test). (C) Plasmid structure provided by the manufacturer (ReGene Biotechnology Co. Ltd., Guangzhou, China). (D) Induction of THP‐1 cells to differentiate into TDMs. Bar = 200 μm. TDMs, THP‐1‐derived macrophages. Figure S3: Co‐Immunoprecipitation assays verifying the direct binding of ANGPT2 to Tie2. Figure S4: Images of tube formation assay and Transwell migration assay processed with ImageJ. Figure S5: MWM test results in 2VO + EMS rats 1 week post‐modeling. (A) Representative image showing the swimming paths in each group rats during the MWM test. (B) Line chart showing the average escape latencies in each group rats during the MWM test. (C) Quantitative analysis of the time spent in target quadrant in each group rats during the MWM test. (D) Quantitative analysis of the number of platform crossings in each group rats during the MWM test. (E) Quantitative analysis of the average swimming speed in each group rats during the MWM test. MWM: Morris water maze. Table S1: Primers used in qPCR in this study. Table S2: Primers used in ChIP‐qPCR in this study. [file CNS-32-e70879-s001.docx]

**Co-Immunoprecipitation (Co-IP)**

293T cells transfected with plasmids encoding Flag-ANGPT2 and/or GST-Tie2 were harvested, washed with pre-chilled PBS, and lysed in ice-cold IP lysis buffer supplemented with protease inhibitors for 30 min. Lysates were centrifuged at 12,000×g and 4 °C for 15–20 min, and protein concentrations in the supernatants were adjusted to 0.2–0.5 mg/mL using a BCA protein assay kit. Magnetic beads were washed three times with pre-cooled equilibration buffer (500 μL per wash). After each wash, beads were isolated using a magnetic rack and the supernatant was discarded. Cell lysates were incubated with anti-Flag or anti-GST antibody with gentle rotation at 4 °C for 30 min to 1 h. Beads were then washed twice with lysis buffer. Following the final wash, 50 μL of 2× SDS-PAGE loading buffer was added to the beads, followed by boiling at 95 °C for 10 min. The eluate was collected by centrifugation at 12,000×g for 5 min and subjected to western blotting analysis.

**
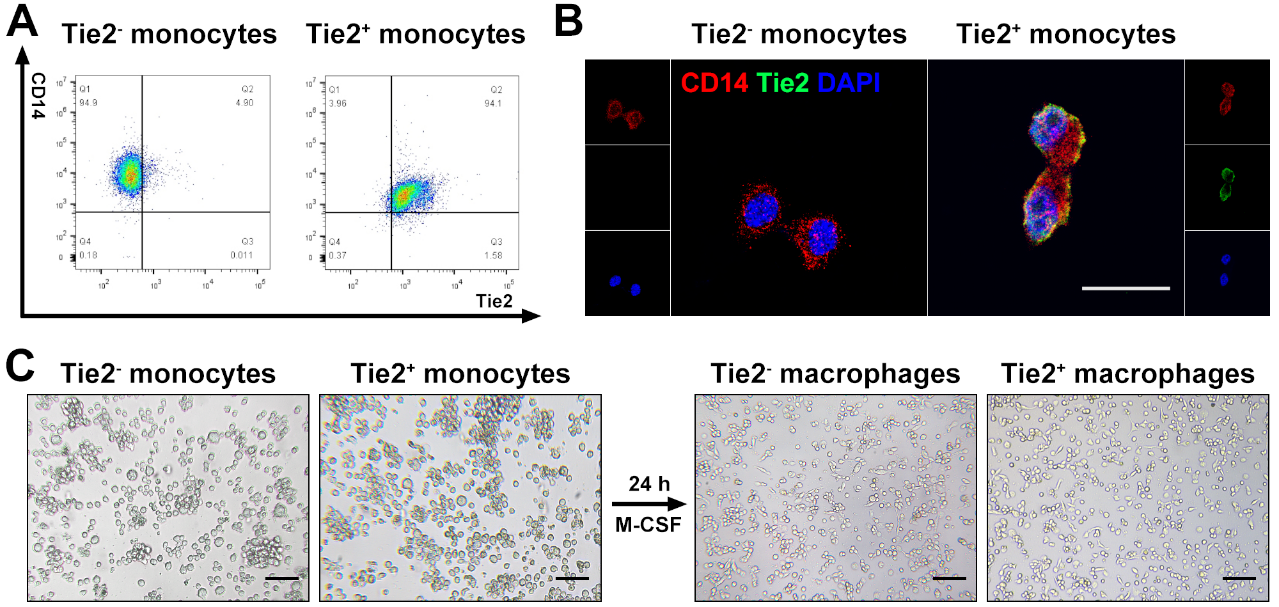
**

**Figure S1.** **Identification of Primary TEMs and TNMs.** (A) Representative flow cytometric dot plot identifying Tie2^+^ monocytes (Tie2^+^/CD14^+^) and Tie2^-^ monocytes (Tie2^-^/CD14^+^). (B) Representative immunofluorescence images identifying Tie2^+^ monocytes (Tie2^+^/CD14^+^) and Tie2^-^ monocytes (Tie2^-^/CD14^+^). Bar = 20 μm. (C) Induction of monocytes to differentiate into macrophages. Bar = 200 μm. TEMs, Tie2-expressing monocytes/macrophages; TNMs, Tie2-negative monocytes/macrophages.


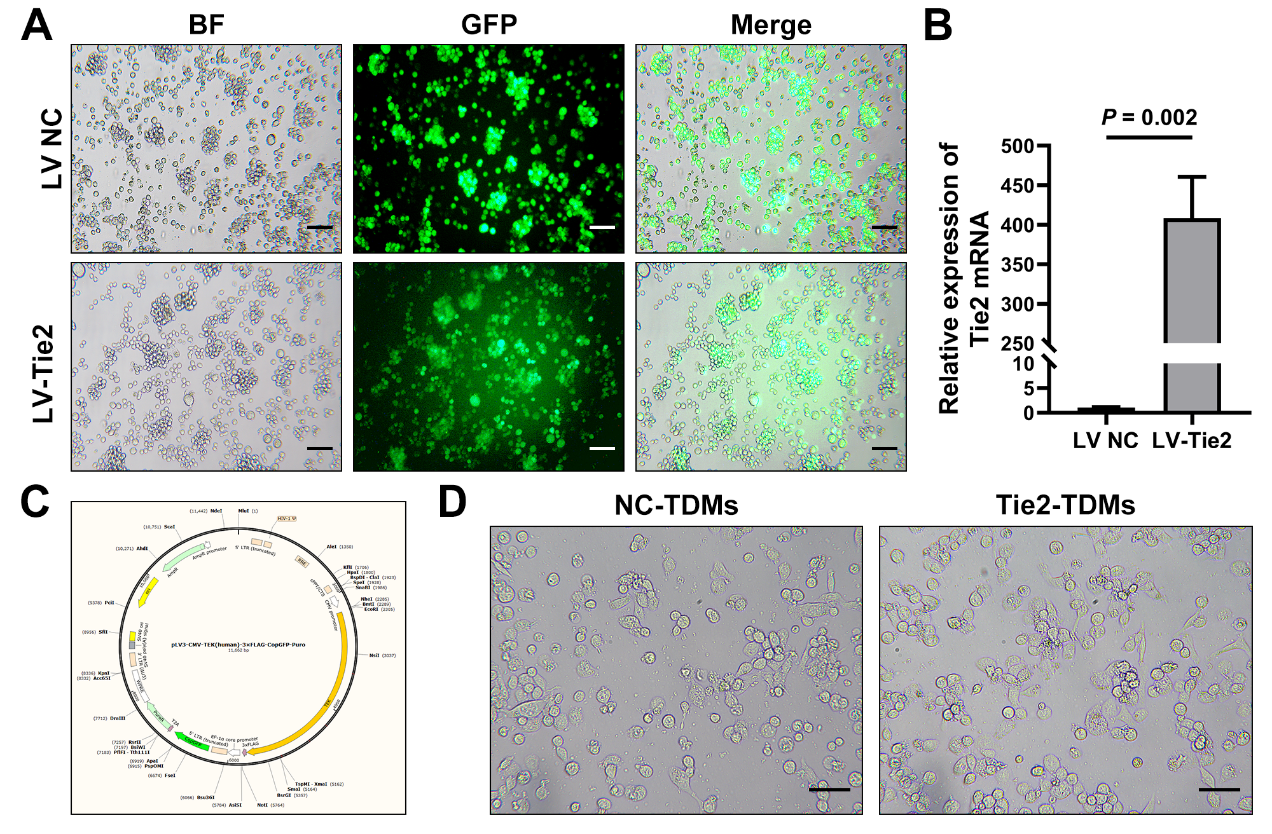


**Figure S2. Preparation of TDMs.** (A) Bright-field and fluorescence images showing the morphology of THP-1 cells after transfection. Bar = 200 μm. (B) Column chart showing the expression level of Tie2 mRNA in THP-1 cells transfected with LV-Tie2 and its negative control (n=3, unpaired t-test). (C) Plasmid structure provided by the manufacturer (ReGene Biotechnology Co., Ltd., Guangzhou, China). (D) Induction of THP-1 cells to differentiate into TDMs. Bar = 200 μm. TDMs, THP-1-derived macrophages.


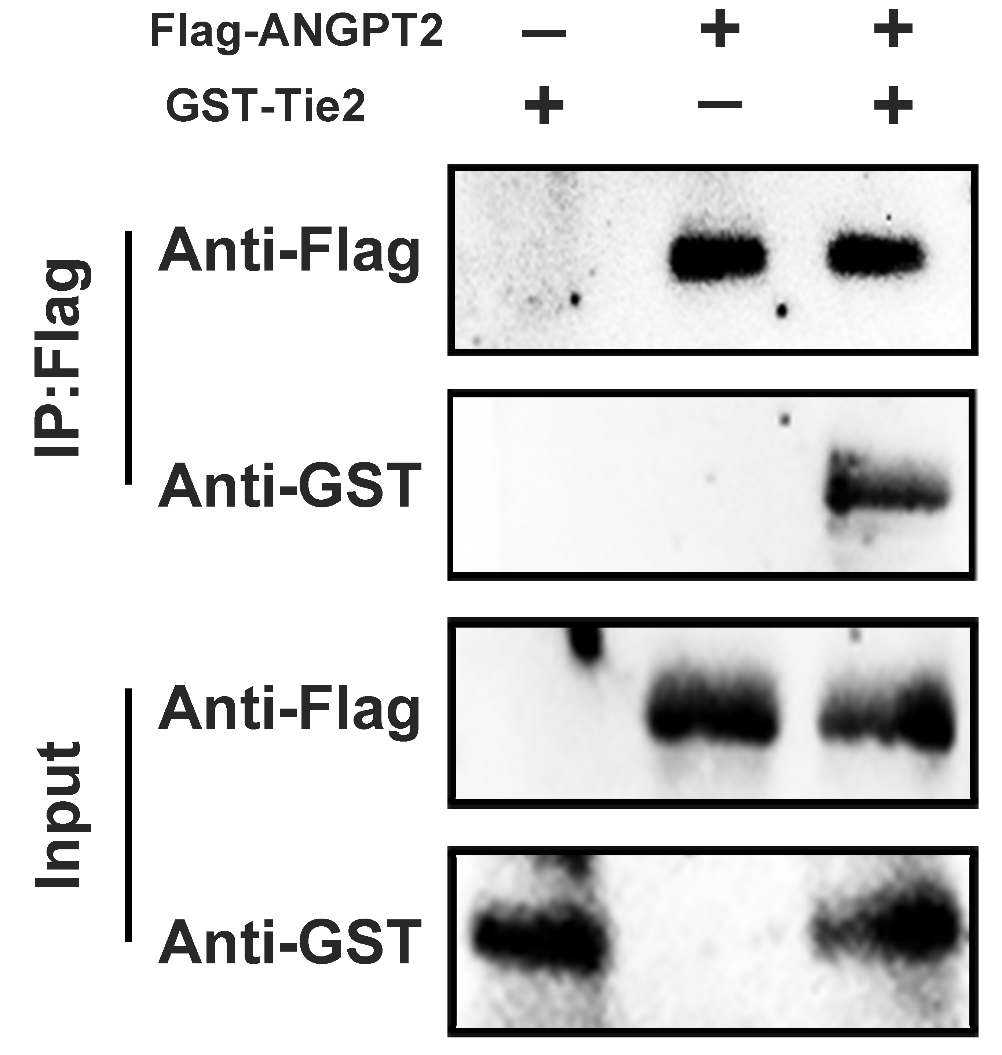


**Figure S3.** **Co-Immunoprecipitation assays verifying the direct binding of ANGPT2 to Tie2.**


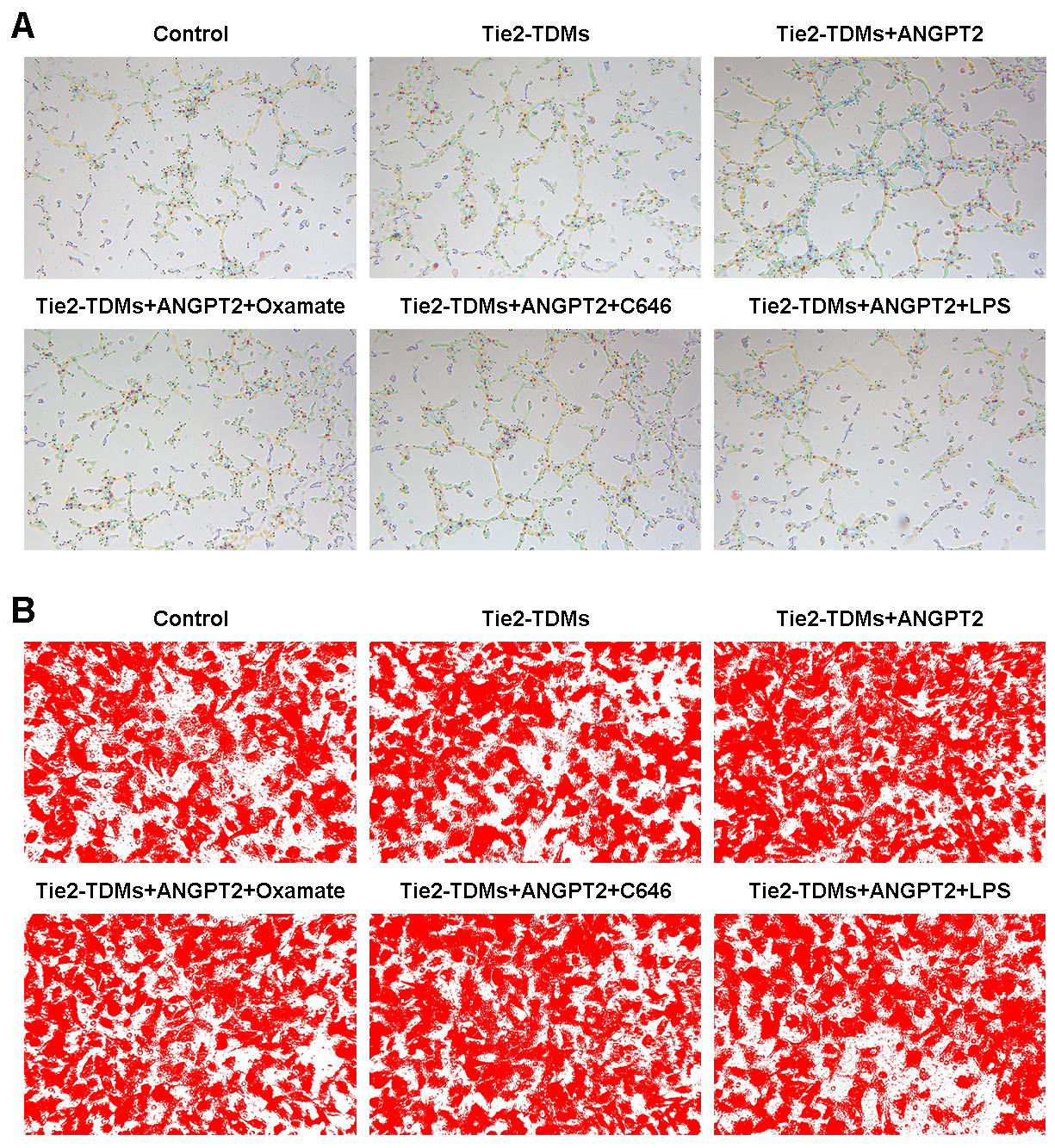


**Figure S4. Images of tube formation assay and Transwell migration assay processed with ImageJ.**


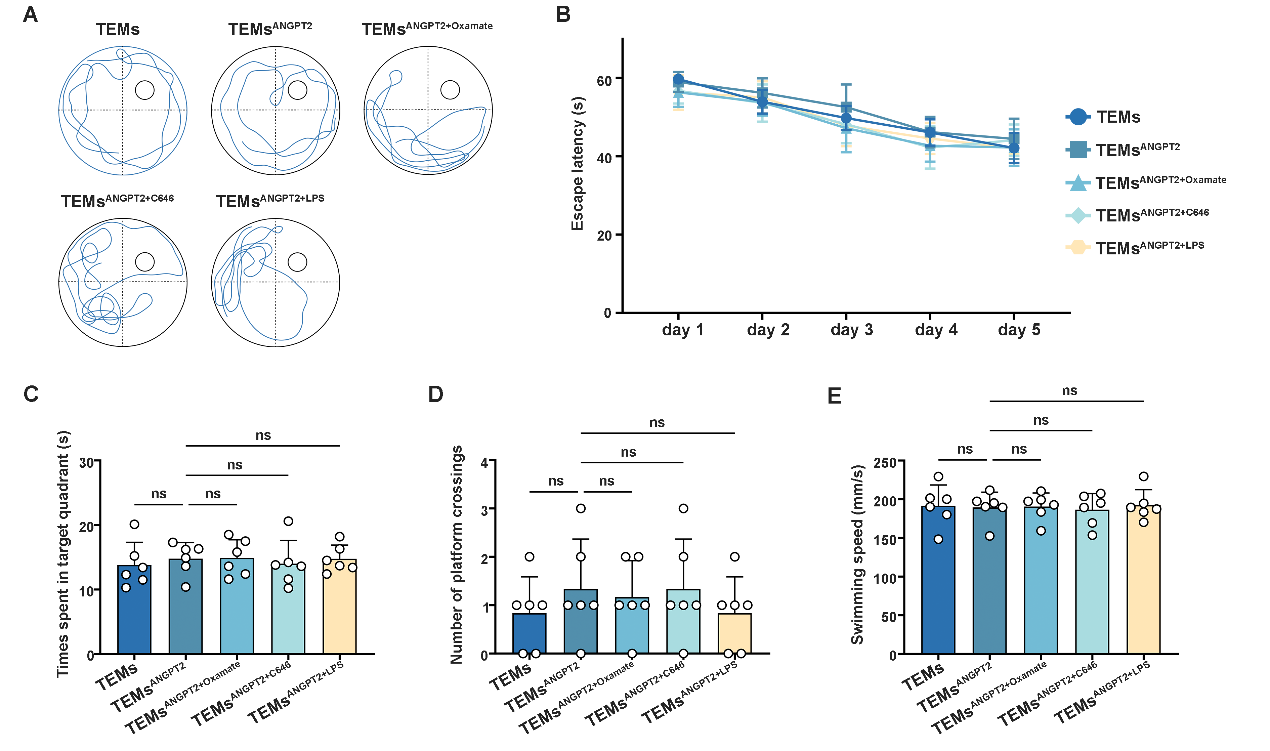


**Figure S5. MWM test results in 2VO + EMS rats one week post-modeling.** (A) Representative image showing the swimming paths in each group rats during the MWM test. (B) Line chart showing the average escape latencies in each group rats during the MWM test. (C) Quantitative analysis of the time spent in target quadrant in each group rats during the MWM test. (D) Quantitative analysis of the number of platform crossings in each group rats during the MWM test. (E) Quantitative analysis of the average swimming speed in each group rats during the MWM test. MWM: Morris water maze.

**Table S1 Primers used in qPCR in this study**

| **Primer** | **Primer Sequence (5' to 3')** | |
| --- | --- | --- |
|  | **F** | **R** |
| *INOS* | CAGGACTCACAGCCTTTGGAC | TGGATGTCGGACTTTGTAGATTC |
| *TNF-α* | GCGACGTGGAACTGGCAGAAG | GCCACAAGCAGGAATGAGAAGAGG |
| *IL-6* | TAGTCCTTCCTACCCCAATTTCC | TTGGTCCTTAGCCACTCCTTC |
| *VEGFA* | CTGGACCCTGGCTTTACTGC | ACTTCACCACTTCATGGGCT |
| *IGF1* | CCACACTGACATGCCCAAGA | GTACTTCCTTTCCTTCTCCTTTGC |
| *EGR1* | GATAACTCGTCTCCACCATCG | AGCGCCTTCAATCCTCAAC |
| *IL-10* | GGACAACATACTGCTAAAGGACTCCT | GCCTGGGGCATCACTTCTAC |
| *MMP-9* | GCAATGTGGATGTTTTTGATGCTATT | CCTGTAATGGGCTTCCTCTATGATT |
| *β-actin* | CTCGACACCAGGGCGTTATG | CCACTCCATGCTCGATAGGAT |

**Table S2 Primers used in ChIP**‒**qPCR in this study**

| **Primer** | **Primer Sequence (5' to 3')** | | | **Genomic** | | **Distance from** |
| --- | --- | --- | --- | --- | --- | --- |
|  | **F** | **R** | **coordinates the TSS** | | | |
| *CD206 promoter* | GAGGTTGTCTGCTGAGGGAC | AGATCAAGCTGCAGGCCATT | chr10:114,793,226 ~ 114,793,365 | | -719 bp ~ -580 bp | |
| *ARG1 promoter* | TGGAAGGGATGTGACAGACG | TGTTCTGGCTCAGCGAACTC | chr6:70,194,316 ~ 70,194,474 | | -928 bp ~ -770 bp | |
| *CD80 promoter* | TACCTCTGCTACCACCTCCT | TCACCACAGTCTCAACCTCC | chr3:119,559,920 ~ 119,560,068 | | 306 bp ~ 156 bp | |
| *iNOS promoter* | AATCCAGGCCTCACTTGACA | GAATCACCTCACAGAAGCGC | chr17:26,501,820 ~ 26,501,987 | | -730 bp ~ -563 bp | |
| *Negative control* | GATGGTGATGGGATTTCCGT | CGTGGTGTAGAGCAGCAGAA | chr1:10,000,050 ~ 10,000,167 | |  | |

TSS, transcription start site.
